# Effects of cold acclimation and dsRNA injections on *Gs1l* gene splicing in *Drosophila montana*

**DOI:** 10.1038/s41598-018-25872-0

**Published:** 2018-05-15

**Authors:** David Hopkins, Tapio Envall, Noora Poikela, Olli T. Pentikäinen, Maaria Kankare

**Affiliations:** 1 University of Jyväskylä, Department of Biological and Environmental Science, Survontie 9, FI-40014 Jyväskylä, Finland; 2 University of Turku, Institute of Biomedicine, Kiinamyllynkatu 10, FI-20520 Turku, Finland

## Abstract

Alternative splicing, in which one gene produce multiple transcripts, may influence how adaptive genes respond to specific environments. A newly produced transcriptome of *Drosophila montana* shows the *Gs1-like* (*Gs1l*) gene to express multiple splice variants and to be down regulated in cold acclimated flies with increased cold tolerance. *Gs1l’s* effect on cold tolerance was further tested by injecting cold acclimated and non-acclimated flies from two distantly located northern and southern fly populations with double stranded RNA (dsRNA) targeting *Gs1l*. While both populations had similar cold acclimation responses, dsRNA injections only effected the northern population. The nature of splicing expression was then investigated in the northern population by confirming which *Gs1l* variants are present, by comparing the expression of different gene regions and by predicting the protein structures of splices using homology modelling. We find different splices of *Gs1l* not only appear to have independent impacts on cold acclimation but also elicit different effects in populations originating from two very different environments. Also, at the protein level, *Gs1l* appears homologous to the human HDHD1A protein and some splices might produce functionally different proteins though this needs to be verified in future studies by measuring the particular protein levels. Taken together, *Gs1l* appears to be an interesting new candidate to test how splicing influences adaptations.

## Introduction

Throughout the genetic and genomic eras it has been increasingly recognised that the way genes are utilised by organisms is as, if not more, important than the number or types of genes present. For instance in eukaryotes, alternative splicing (AS) of genes may explain how complex multicellular organisms can produce many cell types and flexible phenotypes without a corresponding number of genes. In AS, a single gene produce multiple mRNA transcripts (splice variants) mainly by the inclusion or exclusion of different exon and intron regions as pre-mRNA is processed into the mature mRNA^1,2^.

By producing different splice variants, a single gene can produce multiple protein types, with potentially different functions or efficiencies^2^. AS is very common across eukaryotes, as 41–60% of *Arabidopsis thaliana*^3^, 95% of human^4^, and 42–61% of *Drosophila*^5^ genes are suggested to have one or more splice variants. By increasing the diversity of proteins encoded by the genome, AS may not only be extremely important for the proper functioning of organisms^1,2^ but potentially also in the evolution of important adaptations. AS could greatly increase the evolutionary potential of organisms by providing additional sources of variation for selection to act upon^3,6^. Alternatively, AS could provide a mechanism to increase the phenotypic plasticity of organisms faced with stochastic environments, and thus perhaps dampen selection on a trait^7^.

Cold adaptation, especially in the further northern and southern latitudes, is a major determinant of where species can exist^8^. Consequently, understanding such adaptations in detail is a key issue in the face of rapid climate change where species are being pushed out of their native habitats or threatened with extinction, while in other areas warming is opening up habitats to invasion by new species. In plants AS has been demonstrated to play an important role in many adaptations^3,9^, including those associated with temperature^10–12^. However, most animal studies tend to overlook the role of AS in adaptation, especially those carried out with invertebrates^13^, and hence the adaptive role of AS in evolution is less well established, despite some studies demonstrating the adaptive potential of AS in animals^13,14^. For example, Haung *et al*.^13^ specifically found the expression of alternative splices of heat shock proteins (HSP60 and HSP90) in the Pacific Oyster (*Crassostrea gigas*) in response to temperature stress and Ruan *et al*.^14^ showed that switching between two splices of the *Nicotinamide mononucleotide adenylyltransferase* (*Nmnat*) gene in *Drosophila* provides neuroprotection to heat shock stress. Moreover, the temperature dependence of AS seen in some organisms^15–17^ suggests its potential role in stress tolerance.

Often studies on AS and adaptation do not progress beyond the initial step of identifying that AS occurs in biologically interesting genes and fail to test directly the effect of splicing on a particular adaptive trait. To better understand the genetics of adaptation in organisms at the gene level it is necessary i) to find examples of genes where AS is occurring, ii) to investigate what combinations of exons are being expressed, iii) to understand how specific splice variants influence organism’s phenotype and iv) to realize how these phenotypic differences effect organism’s ability to adapt to their environment.

The *Gs1-like* gene has previously been identified as one of the candidate genes impacted in cold acclimation from earlier transcriptome data from northern fly species *Drosophila montana*^18^. Based on our new transcriptome data (see Supplementary Information for more details) this gene also exhibits multiple splicing forms with different expression levels making it an ideal gene to test some of the above-mentioned questions. *D*. *montana* belongs to the *D*. *virilis* species group, and is the most cold tolerant species among 95 *Drosophila* species from three different subgenera^19^. Furthermore, *D*. *montana* populations have evolved multiple adaptions to live on high latitudes close or even above the Arctic Circle in Northern Scandinavia and Alaska.

This study explored the role of AS in *Gs1l* gene in the flies’ adaptation to cold stress in greater depth using multiple genetic methods. *Gs1l* is a protein-coding gene that has previously reported to have wound-healing activity in *Drosophila melanogaster* epithelia and the protein is predicted to have hydrolase activity and function likely as a phosphatase^20^. We first compared the influence of *Gs1l* expression on *D*. *montana* flies from two North American populations (one northern and one southern population) to get an idea of the magnitude of the expression levels in distantly located areas. We did this by altering *Gs1l* expression by injecting adult flies with double stranded RNA (dsRNA) targeting the *Gs1l* gene in cold acclimated and non-cold acclimated flies of two populations. We then tested the cold tolerance of all flies using critical thermal minimum tests (CT_min_) to establish the role of the candidate gene in cold adaptation. During the experiment, the northern population was evidenced to exhibit particularly strong response to injection, so we repeated injections on these flies to investigate the effect of AS on *Gs1l* expression levels. First, we investigated which *Gs1l* splice variants were present in each of the treatments using the RNA extracted from individual flies and by cloning the cDNA products to isolate the different variants. Then the relative expression levels of different gene regions were measured using qPCR to investigate how specific splice variants are responding to cold acclimation and dsRNA injection. Based on this evidence, homology modelling protocol was implemented to predict what protein variants are likely produced from the three different *Gs1l* splices and to speculate on the functionality of the most important splices.

## Results

### Effects of cold acclimation and dsRNA targeting on CT_min_

**Figure 1:**
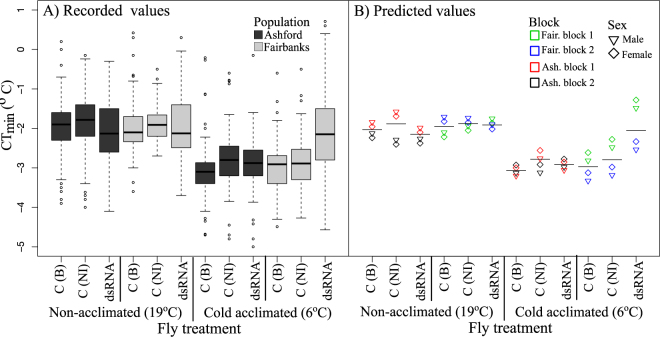
Cold tolerance of the flies from two USA populations. CT_min_ was measured for dsRNA injected flies, “dsRNA” compared to control Ringer buffer injected flies, “C(B)”, and non-injected control flies, “C(NI)”. Flies were also either cold acclimated (6 °C) or maintained at a constant temperature (19 °C) before the cold experiment. (**A**) Recorded CT_min_ values, without block and sex differences shown (error = ±1.5 × IQR). For the full box plot see Supplementary Fig. S1. (**B**) Predicted CT_min_ results, showing the variance caused by fly sex (shapes) and test blocks (colours). Horizontal lines represent the estimated mean of the main treatment classes (error bars =  ± model estimated SEM).

Cold tolerance was quantified using the CT_min_ value, which measures the temperature at which flies are knocked down when subjected to a gradually reducing temperature (see methods for details). Statistical analyses were done by fitting the data to a linear model (LM) that specifically tests the difference between the two controls (buffer injected to non-injected flies) and then the difference between the buffer injected control to the dsRNA injected flies, while also testing for the influence of cold acclimation, sex and experimental block (see methods). A summary of the results are presented in Table 1 and Fig. 1.

**Table 1:** Effect of dsRNA injections to cold tolerance of the flies using CT_min_ measurements.

Model factors + interactions	Estimate	S.E.	t	P
Intercept	−2.24	0.15	−15.40	**<0.001**
Population	0.41	0.19	2.18	**0.029**
Acclimation	−0.69	0.18	−3.83	**<0.001**
C(NI)	−0.17	0.20	−0.81	0.419
dsRNA	−0.14	0.19	−0.74	0.461
Sex	0.11	0.09	1.31	0.189
**Interactions**				
Acclim. Temp. * Population	−0.61	0.24	−2.55	**0.011**
Population * C(NI)	0.14	0.28	0.49	0.624
Population * dsRNA	−0.06	0.25	−0.23	0.822
Acclimation * C(NI)	0.18	0.21	0.83	0.410
Acclimation * dsRNA	0.29	0.20	1.42	0.156
Acclimation * Sex	−0.32	0.12	−2.61	**0.009**
Population * Acclimation * C(NI)	−0.01	0.32	−0.02	0.981
Population * Acclimation * dsRNA	0.69	0.28	2.45	**0.015**
**Nested block**				
Fairbanks Pop./block	−0.39	0.17	−2.31	0.021
Fairbanks Pop./block * Acclimation	0.90	0.18	5.06	**<0.001**
Fairbanks Pop./block * C(B) to C(NI)	0.19	0.24	0.79	0.429
Fairbanks Pop./block * C(B) to dsRNA	0.54	0.20	2.70	**0.007**
Ashford Pop./block	0.28	0.17	1.65	0.098
Ashford Pop./block * Acclimation	−0.35	0.17	−2.03	**0.043**
Ashford Pop./block * C(B) to C(NI)	0.43	0.22	1.95	0.051
Ashford Pop./block * C(B) to dsRNA	−0.01	0.20	−0.03	0.977

LM results testing the difference in CT_min_ values between buffer injected control “C(B)” and non-injected control “C(NI)” flies, between buffer injected controls and dsRNA injected flies “dsRNA”, with flies from two populations, exposed to two different acclimation treatments and tested across two block periods. Significant P values have been bolded. (Intercept = C(B)/non-acclimated/female/Ashford. [+Pop./block 1]).

In this experiment because of practical constraints flies of both gene and cold treatment were tested over two days, representing here as two “test blocks”, for each population. Significant differences in the CT_min_ were found between the blocks for both populations, though these differences were larger in the Fairbank’s population (LM: *P* = 0.021), with difference between blocks in the 6 °C acclimation temperature (LM: *P* < 0.001) and in the dsRNA treatment compared to the buffer injected control (LM: *P* = 0.007). In the Ashford population the differences in block seemed to be only in the non-acclimated flies (Fig. 1, *P* = 0.043). In addition, while there was no overall difference between sexes (*P* = 0.44), there was a modest but significant sex*temperature interaction (LM: *P* = 0.009) suggesting that sexes may have had divergent responses to the acclimation treatment (Fig. 1). Despite the test block and sex differences, as the model predicted values show, the effect of both acclimation and dsRNA treatments (discussed below) were broadly consistent across the block and sexes (Fig. 1B).

As seen in our previous studies^21^, overall acclimation significantly improved the cold tolerance of the flies with an estimated reduction in CT_min_ value of 0.69 °C (LM: *P* < 0.001). Across all population and acclimation treatments, there were no significant differences between the buffer injected and non-injected controls (Table 2). However, there were differences in CT_min_ values between the buffer injected control flies and dsRNA injected flies, but this was only seen in cold acclimated flies of the more northern Fairbanks population (Fig. 1) (LM: Population*acclimation temperature*dsRNA treatment interaction *P* = 0.015). In contrast, in cold acclimated southern Ashford flies dsRNA injection caused little to no difference in CT_min_ compared to the buffer injected control flies (Fig. 1B). Consequently, our results show that dsRNA induced large changes in Fairbanks flies, with injection clearly preventing any increase in cold tolerance that cold acclimation would normally provide, while no such effect was seen in Ashford flies.

**Table 2:** Results of a GLM model testing the expression difference in *Gs1l* exon 2 and exon 3 regions.

Target	Factors & interactions	Estimate	S.E.	t	P
Exon 2	Intercept	−2.56	0.21	−12.48	<0.001
	Acclimation	−0.82	0.29	−2.83	**0.006**
	C(NI)	−0.24	0.29	−0.82	0.417
	dsRNA	0.18	0.28	0.64	0.526
	Acclim. Temp * C(NI)	0.31	0.41	0.76	0.450
	Acclim. * dsRNA	4.12	0.39	10.66	**<0.001**
Exon 3	Intercept	−0.74	0.24	−3.10	0.003
	Acclimation	1.63	0.34	4.85	**<0.001**
	C(B) to C(NI)	−0.35	0.33	−1.08	0.286
	C(B) to dsRNA	0.70	0.32	2.20	**0.031**
	Acclim. Temp * C(B) to C(NI)	−0.03	0.48	−0.07	0.943
	Acclim. Temp * C(B) to dsRNA	−0.82	0.45	−1.83	0.071

The GLM tests for difference between the buffer injected control “C(B)” and non-injected control flies “C(NI)” and between buffer injected control and the dsRNA injected flies “dsRNA”. It also tests for the effect of acclimation and the acclimation*gene treatment interaction. (Intercept = C(B), non-acclimated flies).

### *Gs1l* splice variants in *D*. *montana*

**Figure 2:**
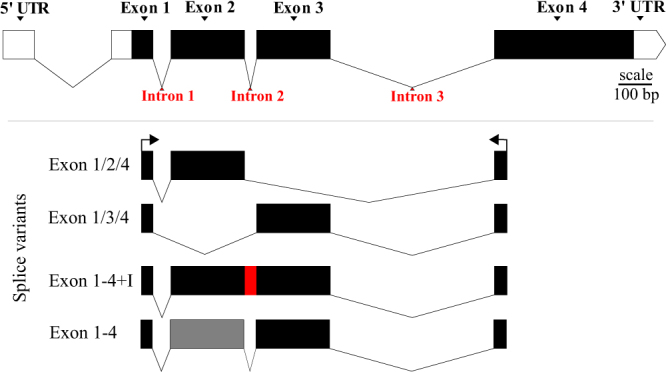
Schematic presentation of the *Gs1l* splice variants. The top sequence gives the presentation of the whole *Gs1l* gene region based on the information from the Korpilahti transcriptome (Supplementary Fig. S4). Splices shown are the four different variants found across Fairbanks flies collected from different treatments for expression analysis in this study. Arrows on the top of the first transcript indicate position and direction of the primers used for the molecular cloning. An intron (red) was retained within the “exon 1–4 + I” variant and there were some differences in exon 2 area of some of the “exon 1–4” splice variants (grey). See Supplementary Fig. S2 for nucleotide and amino acid sequences of the different splices.

By using molecular cloning to isolate *Gs1l* splice variant we identified four distinct splice variants in the Fairbanks fly population (Fig. 2, and see Supplementary Figs S2 and S3 for nucleotide and amino acid sequences and an alignment). Because the primers we used did not cover the whole 5′UTR and 3′UTR regions, we cannot infer differential splicing occurring in these regions. However, we can confirm that there are differences in the coding sequence of different *Gs1l* splices, at least in variants that retain exon 1 and exon 4, with exon 2 or exon 3 spliced in or out. This included both the short variants that contain only one of the exons (2 or 3) at a time (marked as exon 1/2/4 and 1/3/4 in Fig. 2) as well as long variants that contain both of exons 2 and 3 (marked as exon1–4 in Fig. 2). In addition, we found that most of the longer variants (N = 9 of total of 12 sequences) retained the intron region between exon 2 to exon 3 (marked as exon 1–4 + I in Fig. 2). Interestingly, this intron introduces a stop codon in the middle of the sequence but still the rest of the sequence starting from the exon 3 remains the same as in other transcripts (see Supplementary Figs S2 and S3). Above mentioned splice variants found from the Fairbanks population match with those identified in the original transcriptome data collected from Finnish Korpilahti fly population (see Supplementary Fig. S4).

The most common variants found were the shorter transcripts, though this may be also partly due to PCR bias towards shorter products. However, as the two short variants are identical in length, their relative frequency in the cloned colonies should be less prone to PCR bias. Interestingly, the exon 1/2/4 (missing exon 3) splice variants appear to be more common particularly in the cold acclimated flies (see Supplementary Table S1). The commonness of the exon 1/2/4 splice variant in cold acclimated flies suggest that there might be a change in the frequency of splice types expressed in response to cold acclimation. However, because only one female and one male fly of each treatment were tested this observation needs further validation.

Interestingly, as mentioned above within the longer sequences, an intron region between exons 2 and 3 was commonly retained (red section in 1–4 + I in Fig. 2). Of the 12 long transcripts found in the cloned products only 3 were without the intron region and of those the exon 2 was either partially missing or had an additional unknown sequence incorporated, which may suggest these to be artefacts of the cloning process. However, we cannot exclude the presence of longer sequences without the intron 2 region in *D*. *montana*, it may well be that intron 2 has some regulating effects on *Gs1l*, that could also affect protein translation and would be interesting to be investigated in further detail.

### Effects of cold treatment and dsRNA injections on the expression of specific *Gs1l* target regions

**Figure 3:**
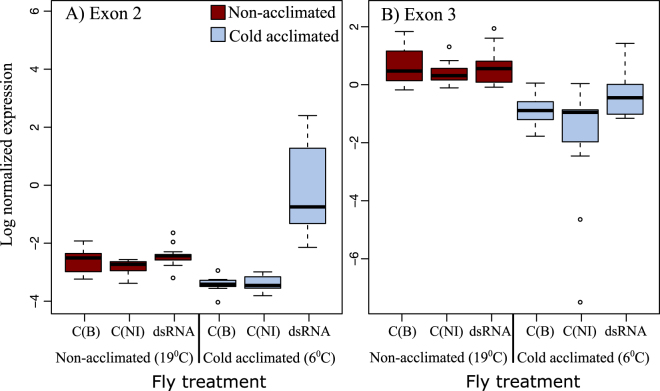
Normalised expression levels of the two regions of the *Gs1l* gene. Flies injected with *Gs1l* dsRNA compared to control flies injected with Ringer buffer “C(B)” and non-injected flies control “C(NI)” in splice variants including exon 2 (akin to T1 and T3–5 in Supplementary Fig. S4) and exon 3 regions (akin to T1–3 in Supplementary Fig. S4) (see also Supplementary Fig. S5 and text for more details). Flies were also either cold acclimated by pre-exposure to 6 °C or non-acclimated as maintained at 19 °C. Error bars = ±1.5 × IQR.

We were interested if the cold treatment and dsRNA injections affected expression levels of different splice variants differently (for simplicity the investigated areas are named here as exon 2 and exon 3 target regions, see Supplementary Fig. S5). To do this, we exposed the flies from the Fairbanks population to the same cold treatments and dsRNA injections as before. Shortly, dsRNA injections were targeted to the area containing a small part of the 5′UTR region, the whole exon1 and part of the exon 2. qPCR was then used to measure how the normalized expression (see methods for details) of the different splice variants including exon 2 (corresponding to the 1/2/4 + 1–4 splice variants) or exon 3 (corresponding to the 1/3/4 + 1–4 splice variants) target regions responded to cold acclimation and treatment. Analysis was performed with a General Linear Model (GLM) in a similar set up as before comparing differences between the two controls and then between buffer injected control flies and flies injected with dsRNA, plus any interactions with fly acclimation conditions. However, in this model the between-sex difference was non-significant so it was dropped from the final analysis (see methods). A summary of the results are presented in Fig. 3 and Table 1.

Overall, both investigated regions showed general expression changes to cold-acclimation, seen in exon 3 region as a large overall decrease in the expression level (1.6 × log fold change, GLM: *P* < 0.001), while in exon 2 region this change was more modest (GLM: *P* < 0.006) and treatment dependant (Fig. 3). Both gene regions also showed similar lack of difference between the buffer injected control flies and the non-injected flies (Table 2). There were differences between buffer injected flies with flies treated with dsRNA in both gene regions (Fig. 3). However, while this effect was both small and general in the exon 3 region (GLM: *P* = 0.031), in the exon 2 gene region expression change was not only specific to the cold acclimated flies and also very large accounting for more than a 4 times log fold increase in the expression level (GLM: dsRNA*acclimation interaction *P* < 0.01). In summary, while there were some expression changes in response to acclimation in exon 3 region and modestly so in the exon 2 region, dsRNA injection appeared to cause strong upregulation of *Gs*1*l* gene only in the exon 2 region, and only in cold acclimated flies.

### Homology modelling to identify splice protein variants

**Figure 4:**
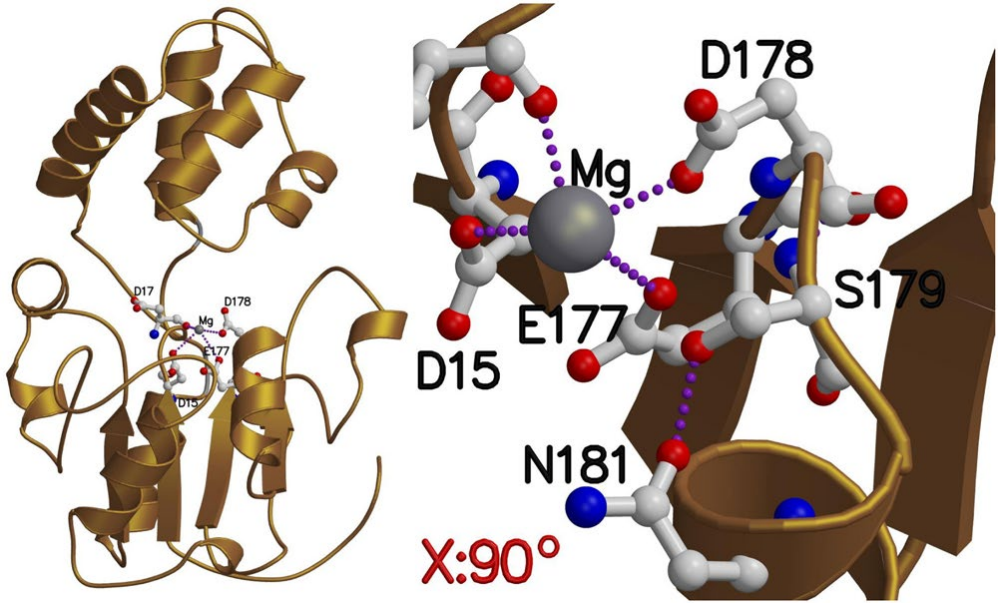
Homology model of the *Gs1l* transcript variant including exon 2. The metal-ion binding site (Magnesium) is formed by similar amino acid residues (shown as ball-and-stick model on left) as in human HDHD1A. The longer variant consists amino acid regions from both shorter variants (including either exon 2 or 3), and if two similar domains would be present they would be connected to positions 25 and 94, which are highlighted with grey coil. The sequence similarity of *Gs1l* variants with HDHD1A is very high, and accordingly, in the vicinity of metal-ion there is only one sequence difference (HDHD1A: A177 (alanine) vs. *Gs1l:* S179 (serine)). In *Gs1l* serine can form hydrogen bonds with neighbouring residues, as shown in the close-up (right, rotated 90 degrees by the x axis).

The best similarity hit for all the three splice variants (those without either exon 2 or 3 and the longer one with all the exons but no intron sequence) was the crystal structure of human pseudouridine-5′-phosphatase, a.k.a. haloacid dehalogenase-like hydrolase domain containing 1 A (HDHD1A). For example, the splice variant including only exon 2 has identical amino acid in 113 positions out of 221 (51%), and additional 37 positions have similar residue. Similarly to HDHD1A, the *Gs1l* enzyme likely binds a metal-ion (Mg^2+^; Fig. 4), which could thus stabilize the substrate position for the catalyzed reaction. Expectedly, practically the whole domain where the metal is bound (amino acid residues 1–22, 86–231; see Supplementary Fig. S3) is conserved, especially at the core and metal-binding site. There are only some point mutations on the amino acids at the surface of this domain, and in one position close to bound metal-ion, where alanine in HDHD1A is replaced with serine in *Gs1l* transcript variants. However, this serine does not likely interfere metal-binding, but rather improves the stiffness of the site by being able to form a hydrogen bond with the neighbouring residues (Fig. 4). Similarly, the domain consisting residues 22–86 is fully conserved in the vicinity of the metal-ion, thus, suggesting that the catalysed reaction can employ the same substrate in all cases. In contrast to the firstly mentioned domain, this domain has more variability, and e.g. sequence stretches 52–59 and 74–83 do not have any conserved residues in any two of the sequences. However, the similarity in residues that surround the catalytic site suggests that the functionality of the *Gs1l* transcripts including either exon 2 or exon 3 should be practically equivalent to that of HDHD1A.

The splice variant containing both exon 2 and 3 has substantially longer sequence than the other two variants as it contains amino acids 25–94 from both shorter variants including either exon 2 or exon 3. Accordingly, as none of the known structures available in the PDB contain similar composition, this transcript cannot be modelled reliably. However, as the connection points, 25 and 94 are in close proximity to each other at the hinge region (Fig. 4. grey coil), it is possible to speculate that this variant could contain two similar domains next to each other, which both would be available upon substrate binding process. However, it is highly likely that this variant would not be fully functional, if even translated into a protein.

## Discussion

Despite the potential importance of alternative splicing (AS) in adaptation, as demonstrated in plants^3,9–12^, there is still a paucity of animal studies and hence the eco-evolutionary role of the AS has not yet been widely tested. Interestingly, a recent cross study comparison concluded that the more complex higher organisms are (as number of cell types) the larger number of AS genes they tended to have^22^. However, the degree to which AS frequency is due to an increase in selection for AS or an increase in genetic drift in larger organisms was unclear^22^. This is why detailed documentation of AS in adaptive genes as well as experimental testing of individual splice effects is important when investigating evolutionary events.

Here we introduce *Gs1l*, a new gene to be associated with adaptive acclimation response to cold stress with interesting AS patterns. Our RNA sequencing (transcriptome) data from *D*. *montana* flies’ from Korpilahti, Finland (62.00°N) evidenced this gene to be differentially expressed between cold acclimated and non-acclimated flies and that different splice variants showed different expression patterns. Interestingly, when investigated in more detail in the northern Fairbanks (64.55°N) population, injections with *Gs1l* dsRNA prevented effect of cold acclimation that normally improves the cold tolerance of the flies. However, there was no such apparent effect of dsRNA injections on the southern Ashford (46.45°N) population.

As Fairbanks flies appeared to exhibit an intriguing response to dsRNA injection, we looked at the flies *Gs1l* expression patterns using qPCR. As detailed above, cold acclimation reduced expression in the exon 2 region (corresponding to the 1/2/4 + 1–4 splice variants), while dsRNA injections on that region dramatically increased expression but only in cold acclimated flies. This dsRNA induced upregulation is in line with the previous observation that *Gs1l* targeted dsRNA injections prevents the cold tolerance flies normally acquire during cold acclimation, meaning higher *Gs1l* expression is maladaptive in cold conditions. On the other hand, dsRNA had little effect on the expression levels of the neighbouring exon 3 region, which is in line with our expectation that the splice variant that includes only exons 1, 3 and 4 but lacks the exon 2 should be unaffected by a dsRNA that targets exon 2.

The difference in the effect of dsRNA injection on the fly cold phenotype suggests that *Gs1l* gene has a somewhat different role in flies from the Ashford as it does in those from the Fairbanks population. This poses a hypothesis that different splice variants might have different effects in different populations as a consequence of either drift causing the loss and/or selection favouring the presence of different splices. This is not so surprising given the large latitudinal and hence also temperature differences between the northern Fairbanks (Alaska) population (64.9°N), with average annual temperature of −2.4 °C (average high 3.3 °C and low −8.1 °C) and southern Ashford (Washington) population (46.8°N), with average annual temperature of 8.0 °C (average high 13.4 °C and low 2.5 °C) (http://www.weatherbase.com/).

### The biology of *Gs1l* and its splices

Currently the exact function of *Gs1l* is largely unknown, but it is a protein-coding gene with predicted hydrolase activity, so it is likely involved in fly metabolism^20^. *Gs1l* is a new gene to be associated particularly with cold stress, though it has been previously linked to other abiotic stresses such as wound healing in *D*. *melanogaster*^20^. In addition, *Gs1l* was found to be one of nine differentially expressed proteins to be associated with post diapause embryonic development of brine shrimp (*Artemia franciscana*)^23^. This latter observation is of particular interest as diapause in *D*. *montana* is intimately linked to the cold tolerance of the flies^24^, also at the gene level^25^.

When moving from the gene expression level to the protein level our homology modelling of the different *Gs1l* sequences from short splices (i.e. either including exon 2 or exon 3) showed that this gene resembles closely human protein Haloacid Dehalogenase-Like Hydrolase Domain Containing 1 (HDHD1), including similar metal-binding sites and hence suggesting similar functionality. The short splice variants of *Gs1l* differ either in sequence between amino acid positions 25–94 or contains a double number of residues in that region. Similar phenomena have been also notified in sequencing results of humans, where four isoforms are reported at the Uniprot database (http://www.uniprot.org/). While the HDHD1 isoform 1 is highly similar to those of both short splices of *Gs1l*, there are also other types of variants: (1) Isoform 2 misses residues 51–93, i.e. the smaller domain is largely missing, (2) Isoform 3 has alternate sequence between residues 171–228, and (3) isoform 4 has additional residues between sequence positions 20 and 21 of isoform 1. Accordingly, from the previously mentioned isoforms most likely only the human HDHD1 isoform 1 has proper functionality.

Human HDHD1 encodes a pseudouridine-5′-phosphatase which is a phosphatase specifically involved in dephosphorylation of Pseudouridine. Pseudouridine is a modified nucleotide present in RNA, including mRNA^26^, tRNAs and ncRNAs^26–29^, and it is thought that the dephosphorylation of pseudouridine 5′-phosphate is an intermediate step in RNA degradation^27^. Catalytic efficiency tests on purified human HDHD1 enzymes suggest that it is substrate specific^27^, in which case the difference seen in *Gs1l* splices may be significant enough to give it different functionality or efficiency. In future work specific activity testing of protein produced from *Gs1l* splices in flies would be insightful and could conclusively test if longer splices, with and without the intron region, are functional at all.

It must also be noted, the haloacid dehydrogenase (HAD) family that *Gs1l* belongs to, is a large protein family with a diverse range of specificity^29^. This makes the predicting the functionality of different splice variants challenging. However, *Gs1l*’s proposed association with RNA regulation certainly make sense when considering the huge range of physiological changes *Drosophila* flies must undergo to survive the many stresses caused by cold exposure (including ionic imbalance, oxidative stress, protein denaturing, changes to membrane fluidity and integrity and inhibition of apoptosis)^30^. Indeed, cold acclimation is known to cause wide spread changes in both the transcriptome^18,30^ and metabolome^30^, in which the regulation would be heavily affected by the ability to regulate their RNA breakdown, especially regulatory RNAs like tRNAs.

### dsRNA induced gene upregulation

An interesting aspect of our results was that injection of the dsRNA into cold acclimated flies did not cause the typical decrease in the expression of the target gene region, known as RNA interference (RNAi), but instead caused a dramatic increase. This kind of up regulatory effect of dsRNA has been recorded before^31–33^ and is often referred to as RNA activation (RNAa)^34^. Indeed, it has become increasingly apparent that small noncoding RNAs (ncRNA), such as dsRNA, have a wide range of regulatory effects when inside cells^35–37^.

While the mechanisms of RNAa are not as well understood as those of RNAi, there is a growing body of evidence that RNAa occurs^33,34,36^. For example, it has been shown that, in at least mammals, mediated by the AGO2 proteins (also used in RNAi) the duplex strand of dsRNA can enter the nucleus to form a RNA-induced transcriptional activation (RITA) complex that stimulates both the initiation and elongation steps of gene transcription causing RNAa^36^.

Our observed RNAa effect is in line with the expectation of the transcriptome results that found *Gs1l* to be down regulated in cold acclimated flies suggesting that higher *Gs1l* expression is likely to be maladaptive as confirmed by the reduced cold tolerance of acclimated Fairbanks flies’ injected with dsRNA. If RNAa is indeed occurring, as far as we know, this maybe one of few published examples of RNAa in Drosophila. The condition dependent action of the dsRNA maybe an important detail in explaining the observed upregulation in *Gs1l* but this needs to be verified in specifically designed experiments using both negative control for dsRNA and multiple fly populations from several geographical areas. Alternative to a direct RNAa action, RNAi action in conjunction with the gene downregulation during flies’ natural cold acclimation response may have been enough to trigger a feedback loop to overexpress the gene in the northern flies. For instance, it is possible to speculate that *Gs1l’s* upregulation may be the result of the gene’s potential role in the regulation of RNAs. If so, this could be an important dynamic of the RNAi/RNAa phenomena. However, it would be interesting to know why the effect of the *ds*RNA injection was inconsequential to Ashford fly cold tolerance.

Regardless of the dsRNA action the key results from our dsRNA injections were firstly that the change in the expression occurred and that this change relates to a difference in cold tolerance that would be expected from the previously seen *Gs1l* expression patterns. Secondly, the effects of dsRNA injections were different between spliced exons. A final interesting result, based on the phenotypic data, was the observed difference between the two fly populations suggesting that *Gsll* gene could be functioning differently between different fly populations. Taken together, *Gs1l* might provide a useful model to investigate the genetic mechanisms of cold tolerance including alternative splicing also in other species.

## Conclusions

To understand the genetic processes that underline adaptations to stress, it is crucial to establish how much important processes like alternative splicing contribute to any one gene’s functionality and evolution. To do this, good gene models in species known to experience environmental stressors are needed and the *Gs1l* gene in *D*. *montana* appears to be a promising new candidate gene to act as one of such models. Different splices of this gene not only appear to have independent impact on cold acclimation but they also propose to have different roles in populations originating from two very different environments. This study highlights the importance to understand how organisms fine-tune expression to environmental stress at the sub gene level.

## Material and Methods

### Selection of the candidate gene to investigate the effect of AS in cold tolerance

The candidate gene for this study was identified using a transcriptome of *D*. *montana* that compiled differentially expressed (DE) genes between cold acclimated and non-acclimated female and male flies population from Korpilahti, Finland. In this study *Gs1l* gene was included at the top 20 DE genes with more than one splice variant in male flies (summarized methods from the transcriptome analyses are given in Supplementary Information). This gene also showed differences between the different variants in the expression levels when sexes and acclimation treatment were compared (Supplementary Fig. S4) and we thus selected it to be investigated further in two geographically distant *D*. *montana* populations from the USA.

### Fly material

*D*. *montana* female flies used in this study were collected in 2016 from 2 mass-bread populations (equalling several hundreds of flies) maintained in the fly laboratory at 19 °C, 65% humidity and on constant light (LL). Each population had been established in the summer 2013 from 15 and 16 individual females collected from the nature from two sites in the USA; Fairbanks (Alaska (64°55′N;147°59′W) and Ashford (Washington, 46°45′N;121°57′W), respectively. All flies were maintained on a yeast-malt diet^38^.

### Producing dsRNA for *Gs1l* gene

Fragment of 395 bp in length from the *Gs1l* gene was first produced with PCR using primers that amplified regions containing a small part of the 5′UTR region, exon1 and part of the exon 2 (Supplementary Fig. S5). PCR product was purified with Genejet Gel Extraction Kit (Thermo Fisher Scientific) and cloned using a CloneJET PCR Cloning Kit (Fermentas, Thermo Fisher Scientific) according to manufacturer’s instructions. Shortly, PCR products were ligated into the vector (pJET1.2/blunt Cloning vector), transformed into *Escherichia coli Zymo JM109* (Zymo Research) cells, which were then colonized on Luria Broth (LB) ampicillin plates. Individual colonies were picked after 16 hours of culturing and cultivated overnight in LB solution including ampicillin using a shaker. The extracted bacterial solutions were used as the template in a second round PCR which was carried out with pJET primers from the cloning kit. PCR products were run in an agarose gel and right size products were selected for the third PCR using again pJET primers but from which the R primer contained T7 promoter sequence at the 5′ end of the primer. PCR products were first purified with GeneJet kit and then used in transcription synthesis of the double-stranded RNA (dsRNA) using the TranscriptAid T7 High Yield Transcription kit (Thermo Fisher Scientific) according to manufacturer’s instructions. Synthesis product were purified using the Tri-reagent (Molecular Research Center, Inc.) and chloroform cleaning, precipitated with isopropanol and quantified using a Nanodrop (Thermo Fisher Scientific, UK) and agarose gel staining.

### Fly collecting, dsRNA injections and cold tolerance testing of the flies

Newly emerged flies (≤1 day old) were collected from the Fairbanks and Ashford mass-bread populations, separated by sex and kept at 19 °C in constant light (LL) to prevent flies to enter to diapause. After 16 days half of both females and males (20–30 flies) were subjected to the cold acclimation treatment for 5 days at 6 °C (as done in previous studies^20^) in LL and the other half remained at 19 °C in LL. At the age of 19 days both cold acclimated and non-acclimated (cold control) flies were collected from the chambers, anesthetized with CO_2_ and injected with 436 ng of dsRNA targeting to *Gs1l* gene or with Ringer buffer (injection control). We also collected non-injected flies and treated them exactly the same way as injected flies i.e. they were anesthetised with CO_2_ and moved under the microscope and back to tubes but without injections. To prevent any effects CO_2_ anesthesia might have on the flies, only 5 flies were injected at a time, making anesthetization time to be less than 2 minutes. After injections, flies were moved back to the maintenance chambers for two more days.

At the age of 21 days, all flies had their cold tolerance quantified by measuring their critical thermal minimum (CT_min_) using a Julabo F32-HL device. CT_min_ is the temperature at which flies are knocked down (fall down and show no muscle activity) when subjected to a gradually reducing temperature. In this experiment, flies were placed in sealed glass tubes (3 individual flies per tube in separated sections = 24 flies in total per test) and submerged into a 30% glycol solution, which was then chilled at the rate of 0.5 °C per minute from a starting point of +19 °C down to flies’ thermal chill limit. This was performed over two-test periods for each fly population to give overall 6–27 replicates (median N = 16, actual sample sizes are given in the Supplementary Table S2) for the different treatment group combinations including sex, population, acclimation condition and dsRNA.

Significance in the differences in CT_min_ results among the sex, dsRNA treatment, acclimation condition and population, and their interactions, were tested by fitting data to a linear regression model (LM) using the lm() function in base R (https://www.R-project.org/). LM significance values for dsRNA treatment were presented as a sequential analysis by using the buffer control treatment as the intercept of the model. In doing this first the difference between the buffer injected and no-needle controls were tested to establish the influence of injection had on flies, then the difference between buffer injected control and dsRNA injected flies was presented to establish the effect dsRNA had on flies. In addition, the LM model predicted values were computed to aid the interpretation of differences between all treatments and replicates, using the predict() function in base R (https://www.R-project.org/). Due to practical constraints testing for each population was done on two separate occasions. Within the LM model, these two test blocks were treated as a fixed factor nested within fly population. As we were not specifically interested in sex differences in this study, gender and any interactions with gender was included in the models only if they explained a significant amount of the variation; which here was the sex by acclimation temperature interaction.

### Fly collecting for the RNA extractions and cDNA synthesis

To investigate the levels of expression in different *Gs1l* gene regions exactly at the time of the knock down, we used injected flies from the Fairbanks population and used the same lot of dsRNA for injections and same acclimation treatments as in the above mentioned cold experiment. Immediately after they were knocked down flies were placed in individual Eppendorf tubes, immersed into liquid nitrogen and saved in −80 °C freezer for RNA extractions. We did not use the CT_min_ data from these experiments as the collecting of the flies precisely at the right time made checking the knock down temperature unreliable. We used qPCR to measure the relative expression of the separate *Gs1l* gene regions including exon 2 and exon 3 target regions. RNA extractions were performed using ZR Tissue & Insect RNA MicroPrep Kit with DNAse treatment (Zymo Research^®^), using 5–7 successful replicates from each of the treatments. RNA purity was checked with NanoDrop® ND-1000 spectrophotometer (Thermo Fisher Scientific) and concentration with The Qubit® 2.0 Fluorometer and hsDNA kit (Thermo Fisher Scientific) and cDNA was generated using equal concentrations of RNA (100 ng/µl) and iScript Reverse Transcription Kit (Bio-Rad Laboratories^®^).

### Identification of variants with different exon content using molecular cloning and Sanger sequencing

To identify the *Gsl1* variants with different exon content present in the Fairbank flies, molecular cloning was used for the above mentioned cDNA collected originally from the cold tolerance tests. The *Gs1l* gene was first amplified from cDNA by PCR, using a forward primer positioned at end of the first exon (“exon 1” in Fig. 2) and a reverse primer positioned on the last exon (“exon 4” in Fig. 2). Next, 1 µl of single PCR product from each gender, acclimation/control condition and dsRNA treatment combination was cleaned using GeneJET Gel Extraction Kit (ThermoScientific™) and ligated into the vector (pJET1.2/blunt Cloning vector). Samples were then transformed into plasmids containing ampicillin resistant gene using CloneJET PCR Cloning Kit (ThermoScientific™) according to manufacture’s instructions. 25 µl of *E*. *coli* (strain JM109) were exposed to 2.5 µl of each plasmid and plated to Luria Broth plates containing 100 µg/ml of ampicillin. After that the plates were incubated for 17 hours at 37 °C, individual bacterial colonies were picked, placed in well of 50 µl nuclease free water and lysed by boiling at 90 °C for 10 minute. 1 µl of each colony sample (96 in total) where then amplified by PCR using the pJET 1.2 primers and run on the 1.5% agarose gel to check for successful insertion and size distribution of the different products. This showed that the majority of *Gs1l* products in successfully cloned colonies were of a short length (i.e. contained either exon 2 or 3; just under 500 bp long) while 12 of the samples, across all flies, were of a longer length (including both exon 2 and 3; just over 600 bp long, see Supplementary Table S1 for information about sequenced clones).

For each original fly sample all the long products and 5 randomly selected short products were chosen for Sanger sequencing and sequencing runs were carried out with ABI 3130xl Genetic Analyzer (Thermo Fisher Scientific) using pJET1.2 F and R primers. Sequence analysis and alignments were compiled using Sequence Analysis software (Thermo Fisher Scientific) and BioEdit sequence alignment editor version 7.2.5^39^ to collect detailed nucleotide information from all the different splice variants.

### Quantitative real time PCR (qPCR)

All primers for qPCR were designed based on *D*. *montana* genomic sequences^40^ using Primer3 (primer3.ut.ee) and NetPrimer (www.premierbiosoft.com/netprimer) programs (for primer sequences see Supplementary Table S3). qPCR mix contained 10 µl of 2× Power SYBR Green PCR Master Mix (Bio-Rad Laboratories), 0.3 µM of each gene-specific primer and 1 µl of cDNA solution. Cycling conditions in Bio-Rad CFX96 instrument were: 3 min. at 95 °C, 10 sec at 95 °C, 10 sec at annealing temperature 55 °C (except for the *Gs1l* exon 2 primer which was 66 °C) and 30 sec. 72 °C (repeated 40×), followed by melting curve analysis (65 °C–95 °C) for amplification specificity checking. Each run included 3 technical replicates for each sample and the final threshold value (Cq) was defined as mean of the technical replicates that produced good quality data.

The expression of exon 2 and exon 3 regions of *Gs1l* gene were measured using qPCR (Supplementary Fig. S5). In addition, expression levels of 4 commonly used control genes (*18 S Ribosomal RNA* (*18 S*), *Elongation factor 1 alpha 100E* (*Ef1α100E)*, *Ribosomal protein* 32 (*RpL3*2) and *alpha-Tubulin 2* (*α-Tub2*)) were measured to be used for the normalisation of the *Gs1l* targets. However, as only *Tub2* had an expression pattern that was consistent across all treatments and/or gender combinations to meet proper qPCR requirements^41^ (Supplementary Fig. S6), we thus normalised the gene expression using a data driven normalization algorithm in NORMA-Gene program^42^. For comparison, normalisation was also performed using the ∆∆Ct normalisation method^43^ using the real efficiency value of the *Tub2*, which yielded similar results (Supplementary Fig. S7).

Differences in the expression levels for each gene region between the factor groups of fly gender, dsRNA injection or control treatment, acclimation conditions, and their interactions were modelled using generalised liner model (GLM), with a gamma distribution and log link. This was done using the glm() function in base R (https://www.R-project.org/). Again the effect of gene treatment was tested as sequential analysis in which the difference between buffer injected control and non-injected control and then the buffer injected control and the dsRNA treatment. Also as before, fly gender was only included in the final model if it explained a significant amount of the variation, which was not the case for either target regions. This gave a final sample size ranging from 11 to 16 for each fly treatment acclimation condition combination (5 to 10 for each fly treatment, acclimation condition and gender combination, see Supplementary Table S4 for exact sample sizes).

### Homology modelling to identify protein models from different splice variants

We used three different *Gs1l* variants, namely both short variants including either exon 2 or exon 3 (exon 1/2/4 and 1/3/4) and the long variant including both exons 2 and 3. All the variants were used separately in Blast search to identify similar proteins to better understand the 3D structure of each of these variants using both Blast in Uniprot (www.uniprot.org), and the protein data bank (PDB; www.rcsb.org). The best hit for all three transcripts was the crystal structure of human pseudouridine-5′-phosphatase, a.k.a. haloacid dehalogenase-like hydrolase domain containing 1 A (HDHD1A; PDB code: 3L5K; Uniprot: HDHD1_HUMAN). Accordingly, the crystal structure of HDHD1A offered a plausible template for homology modelling of different *Gs1l* transcripts. Next, the sequence alignments of all the transcripts and the crystal structure of HDHD1A were made with Malign in Bodil Modeling Environment^44^ by employing structure-based matrix^45^ (STRMAT110) with gap formation penalty of 60. Based on formed sequence alignment, models of transcripts were built with Modeller v9.16^46^.

### Data availability

All data generated or analysed during this study are included in this published article (and its Supplementary Information and Dataset files).

## Electronic supplementary material

Supplementary Information 1

Supplementary Dataset 1
