# Supplementary material for: Effects of cold acclimation and dsRNA injections on Gs1l gene splicing in Drosophila montana
Source: Sci Rep. 2018 May 15;8:7577. doi: 10.1038/s41598-018-25872-0 (PMC5953924; doi:10.1038/s41598-018-25872-0)
Supplement: Supplementary file 1 — Supplementary Information 1 [file 41598_2018_25872_MOESM1_ESM.pdf]

Supplementary Information: Effects of cold acclimation and dsRNA injections on *Gs1l* gene splicing in *Drosophila montana*”

David Hopkins[1], Tapio Envall[1], Noora Poikela[1], Olli T. Pentikäinen[1,2] and Maaria Kankare[1*]

[1]University of Jyväskylä, Department of Biological and Environmental Science, Survontie 9, FI-40014, Jyväskylä, Finland

[2]University of Turku, Institute of Biomedicine, Kiinamyllynkatu 10, FI-20520 Turku, Finland

*Corresponding author: s.maaria.kankare@jyu.fi, Tel. +358(0)408054332

Supplementary information: Description of the data analysis of the transcriptome data from which only the information of *Gs1l* gene was used in this study.

Sample collection

21 days old cold acclimated and non-acclimated (control) *D. montana* female and male flies (treatments as described in the methods of the manuscript) were collected from the Korpilahti (Finland, 62°00'N, 25°34'E) mass-bread population for RNA extractions (done in the same way as described in the methods). We used three replicates each consisting of pooled sample of 3 flies and library preparation was carried out according to Illumina TruSeq® Stranded mRNA Sample Preparation Guide. Samples were sequenced with Illumina TruSeq technology with 150 bp forward and reverse reads using Finnish Functional Genomics Center (FFCG) in Turku (https://www.btk.fi/). For each of the 12 samples, a total of 60-90 M paired-end reads were sequenced.

Quality checking and trimming

The quality of raw reads was checked with FastQC 0.11.4[1]. Illumina TruSeq Adapters ('Illumina adapter sequences' 2009) were detected and removed with CutAdapt 1.9.2.dev0 (with Python 2.7.6)[2]. Quality was rechecked with FastQC, after which reads were trimmed to length of 140 bp with Trimmomatic 0.35[3]. Next, bases falling below a quality score 30 were cut from the beginning (LEADING:30) and the end of the read (TRAILING:30). Finally, 17 bp wide reading frame, "a sliding window", was slid through the read starting from the 5'-end, and when average quality within the sliding window dropped below 19, the rest of the read was discarded (SLIDINGWINDOW:17:19). After this step, reads shorter than 85 bp were discarded (MINLEN:85), in order to minimize expression bias by short, aggressively trimmed reads[4]. On average, a total of 6 % of the reads were lost in the trimming steps.

Mapping the reads to the genome

Before mapping, a bowtie2-index[5] was built. Then Tophat 2.0.13[6] was used for mapping with --read-edit-dist and –N 4 with respect to *D. montana* genome with its annotation, for each sample in each lane separately (12 samples x 5 lanes = 60). Following this read mapping step, the mapping percentages were inspected and when no discrepancies were detected the mapped reads files of different lanes were merged within samples with SAMtools merge 1.3[7]. Mapping success was 70–81 %.

Constructing the annotation

The annotation used in this study had been constructed with RNA-seq reads from two previous studies[8,9]. To build a custom transcript-annotation, where the majority of transcripts from the RNA-seq experiment were included, Cufflinks 2.2.1[10] was used as follows. Aligned reads and annotation served as inputs to Cufflinks and it was run separately for each sample. Then the sample-specific annotations were combined to construct a common annotation for all the samples with cuffmerge 2.2.1. The few transcripts whose direction (+ or -) Cufflinks could not determine (Cufflinks symbol) were converted to forward (+) direction for convenience. Transcripts having non-canonical bases at the splicing sites were assumed to be technical noise rather than result of actual non-canonical splicing. Thus, these transcripts were filtered out using gffread, a program included in the Cufflinks package, with canonical requirement –N and including the genome, option –g. Also, strandedness of the transcripts was checked and transcripts overlapping features in the original annotation but going to the different direction were discarded. The overlap between cufflinks-transcripts determined from

the RNA-seq data and genes in the original annotation was inspected with bedtools 2.17.0[11], and a number of transcripts spanning two or more annotated genes were found. Those were assumed to be of non-biological origin and [12,13].

A final step in constructing the transcript-annotation was removal of rRNA transcripts. The rRNA sequences were obtained from *D. virilis* ("dvir-all-miscRNA-r1.2.fasta" from FlyBase [14] and blasted (v. 2.3.0) with blastn (provided by NCBI Resource Coordinators, ww.blast.ncbi.nlm.nih.gov) against the transcript-annotation. rRNA transcripts were then removed from the annotation. This final transcript-annotation was then used as a reference to remap quality-trimmed reads to the genome with Tophat with the same parameters as before. Then, the potential genes were extracted from this transcript-annotation with gffread, forming gene-annotation to be used for counting reads mapped to genes. Results using the updated annotation were compared to those conducted with the original annotation to assess the impact of the new annotation. Finally, a reference for transcript calculation was constructed from the updated annotation with RSEM 1.3.0[15] with rsem-prepare-reference.

Counting the reads and filtering

Read counts for genes were obtained separately using HTSeq (v. 0.6.1)[16] with option --stranded=no, using the corresponding prepared annotation described above. Read counts for transcripts were obtained with RSEM (v. 1.3.0), using trimmed reads and prepared reference as inputs. The genes and transcripts were filtered according to the read counts removing those with negligible expression and those whose expression was limited to one sex. A generalized, modified approach of maximum-based methods was chosen: the threshold was counts per million (cpm) greater than 1 for at least two samples of each sex and cpm greater than 0 for every sample. Features with lower expression were excluded from further analysis. This relatively harsh filtering was applied in order to improve false discovery rate (FDR) control[17].

Differential expression (DE)

Filtered read counts were used as inputs for DE analysis between control/acclimated and male/female flies. Differential expression was calculated using R with Bioconductor package edgeR[18]. First, samples were grouped according to their gene counts with principal coordinate analysis, including all the (filtered) genes (edgeR function plotMDS with top = Inf), to visualize and confirm the split between sexes and acclimation treatment. To check the quality of data, between-sample variance i.e. dispersion in gene expression was then examined with respect to expression levels and visualized with edgeR function plotBCV with default parameters. Read counts were normalized using the TMM (trimmed mean of M-values) method[19]. Then a generalized linear model (GLM) with a negative binomial distribution was fit to the data. Factors included were sex and treatment (cold acclimated or control). A within-gene between-sample variance parameter, tag wise dispersion, was individually estimated for each gene. Tag wise dispersion was then used in a GLM likelihood ratio test to determine if a gene was significantly down- or upregulated between cold acclimated and control flies, i.e. DE in cold acclimation. P-values from the GLM likelihood ratio test were multiple tests corrected using Benjamine and Hochberg's equation to control false discovery rate (FDR)[20] with a significance threshold level of < 0.05.

For transcripts, the DE analysis in cold acclimation was similar to the gene level analyses described above, but after calculating p-values for individual transcripts with edgeR, the function perGeneQValue from R-package DEXseq 3.5[21,22] was used to convert significance values to the gene-level, to avoid overestimating the significance values of genes with more transcripts. This

approach captured genes with differential transcript usage[23], but it also included genes with differential expression at the gene level, without any differential transcript usage.

References

1. Andrews S. FastQC: a quality control tool for high throughput sequence data. Available online at: http://www.bioinformatics.babraham.ac.uk/projects/fastqc (2015).

2. Martin M. Cutadapt removes adapter sequences from high-throughput sequencing reads. *EMBnet. journal* **17**, 10–12 (2011).

3. Bolger A.M., Lohse M. & Usadel B. Trimmomatic: A flexible trimmer for Illumina sequence data. *Bioinformatics* **30**, 2114–2120 (2014).

4. Williams C.R., Baccarella A., Parrish J.Z. & Kim C.C. Trimming of sequence reads alters RNA-Seq gene expression estimates. *BMC Bioinformatics* **17**, 103 (2016).

5. Langmead B. & Salzberg S.L. Fast gapped-read alignment with Bowtie 2. *Nat Methods* **9**, 357–359 (2012).

6. Trapnell C., Pachter L. & Salzberg S.L. TopHat: Discovering splice junctions with RNA-Seq. *Bioinformatics* **25**, 1105–1111 (2009).

7. Li H., Handsaker B., Wysoker A., Fennell T., Ruan J., Homer N., Marth G., Abecasis G. & Durbin R. The Sequence Alignment/Map format and SAMtools. Bioinformatics **25**, 2078–2079 (2009).

8. Parker D.J., Vesala L., Ritchie M.G., Laiho A., Hoikkala A. & Kankare M. How consistent are the transcriptome changes associated with cold acclimation in two species of the *Drosophila virilis* group? *Heredity*: 1–9 (2015).

9. Parker D.J., Ritchie M.G. & Kankare M. Preparing for Winter: The Transcriptomic Response Associated with Different Day Lengths in *Drosophila montana*. *G3:* **6**, 1373–1381 (2016).

10. Trapnell C., Williams B.A., Pertea G., Mortazavi A., Kwan G., Baren M.J. van, Salzberg S.L., Wold B.J. & Pachter L. Transcript assembly and quantification by RNA-Seq reveals unannotated transcripts and isoform switching during cell differentiation. *Nature Biotechnol.* **28**, 511–515 (2010).

11. Quinlan A.R. & Hall I.M. 2010. BEDTools: A flexible suite of utilities for comparing genomic features. *Bioinformatics* **26**, 841–842 (2010).

12. Palmieri N., Nolte V., Suvorov A., Kosiol C. & Schlötterer C. Evaluation of Different Reference Based Annotation Strategies Using RNA-Seq - A Case Study in *Drososphila pseudoobscura*. *PLoS ONE* **7** (2012).

13. Palmieri N., Nolte V., Chen J. & Schlötterer C. Genome assembly and annotation of a *Drosophila simulans* strain from Madagascar. *Mol. Ecol.Res.***15**, 372–381 (2015).

14. Tweedie S., Ashburner M., Falls K., Leyland P., McQuilton P., Marygold S., Millburn G., Osumi-Sutherland D., Schroeder A. & Seal R. FlyBase: enhancing Drosophila Gene Ontology annotations. *Nucleic Acids Res.* **37** (2009).

15. Li B. & Dewey C.N. RSEM: accurate transcript quantification from RNA-Seq data with or without a reference genome. *BMC Bioinformatics* **12**, 323 (2011).

16. Anders S., Pyl P.T. & Huber W. HTSeq-A Python framework to work with high-throughput sequencing data. *Bioinformatics* **31**, 166–169 (2015).

17. Soneson C., Matthes K.L., Nowicka M., Law C.W. & Robinson M.D. Isoform prefiltering improves performance of count-based methods for analysis of differential transcript usage. Genome Biology **17**, 12 (2016).

18. Robinson M.D., McCarthy D.J. & Smyth G.K. edgeR: A Bioconductor package for differential expression analysis of digital gene expression data. *Bioinformatics* **26**, 139–140 (2010).

19. Robinson M.D. & Oshlack A. A scaling normalization method for differential expression analysis of RNA-seq data. *Genome Biol.* 11 (2010).

20. Benjamini Y. & Hochberg Y. Controlling the False Discovery Rate: A Practical and Powerful Approach to Multiple Testing. *J R Stat Soc Series B Stat Methodol.***57**, 289–300 (1995).

21. Anders S., Reyes A. & Huber W. Detecting differential usage of exons from RNA-seq data. *Genome Research* **22**, 2008–2017 (2012).

22. Reyes A., Anders S., Weatheritt R.J., Gibson T.J., Steinmetz L.M. & Huber W. Drift and conservation of differential exon usage across tissues in primate species. *PNAS* **110**, 15377–15382 (2013).

23. Soneson C., Love M.I. & Robinson M.D. Differential analyses for RNA-seq: transcript-level estimates improve gene-level inferences. *F1000 Research* **4**, 1521 (2015).

Supplementary figures and tables for "Effect of cold acclimation and dsRNA injections on *Gs1l* gene splicing in *Drosophila montana*"

Fig. S1

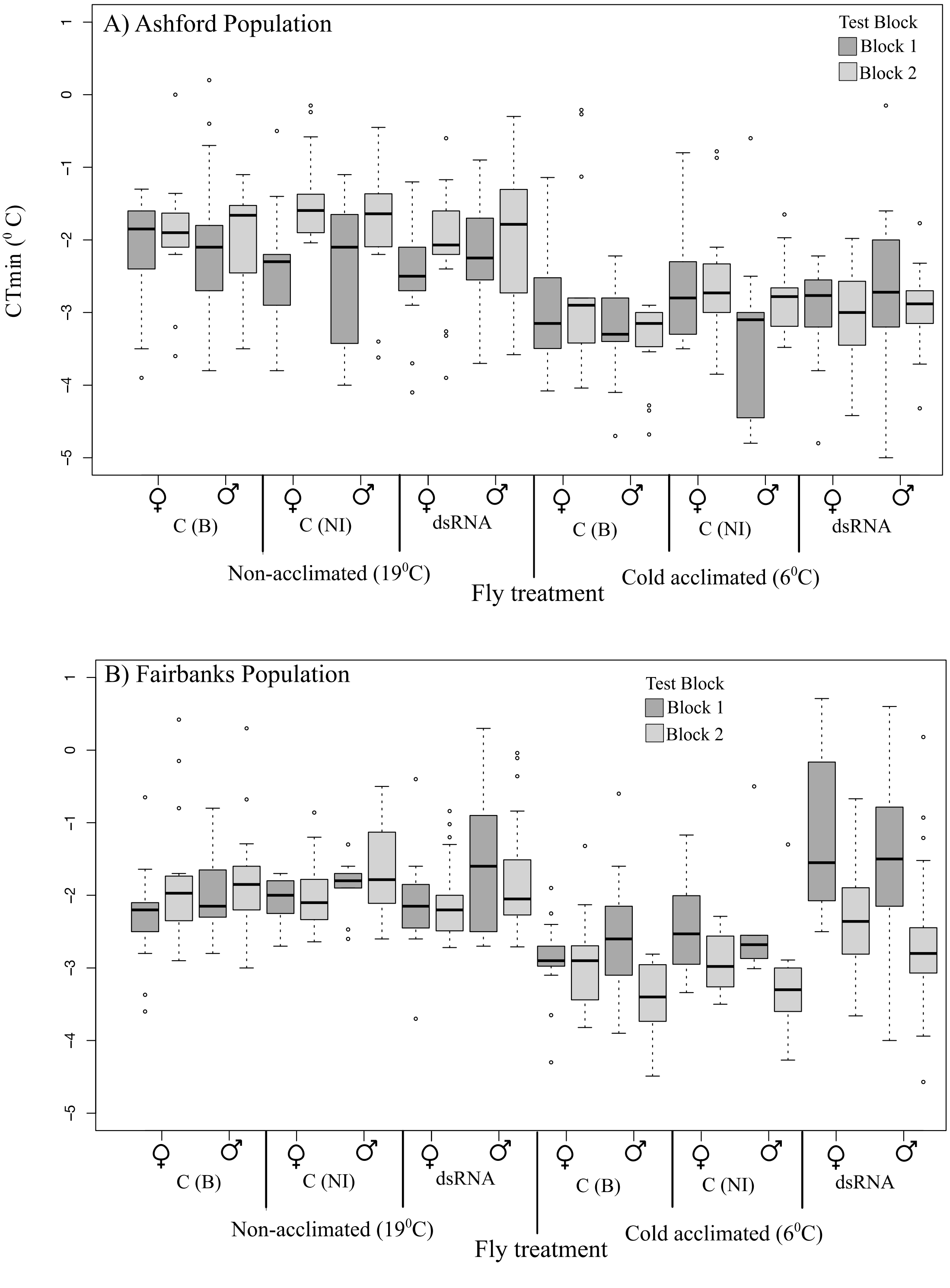

Figure S1. Full break down of the cold tolerance data showing treatment and block and sex differences in *D. montana* flies. CT_{min} was measured for dsRNA injected flies, "dsRNA" compared to control Ringer buffer injected flies, "C(B)", and non-injected control flies, "C(NI)". Flies were also either cold-acclimated (6°C) or maintained at a constant temperature (19°C) before the cold experiment. A) flies from Ashford population, B) flies from Fairbanks population.

Fig. S2

>Exon 1/2/4, exon 3 missing (T4)
ATGGCTCAGAAGGTATTGCGAAATGTGACGCACTGCATCTTTGACATGGATGGCCTGCT
GCTGGACACTGAGACCCTCTACACGAAGGCCGCCCAAATGGTGCTCGATCCGTATGGCA
AGACGTACACGTTCGATGTGAAGCAGCAGATAATGGGTCTGCAGACGCGTCCGGTGGC
CGAGTTCATGATTAAATGCTACGACCTACCGATGACTTGGGAAGAGTACGCCAAACAAC
AGACGGACAATGCACGCGCCCTCATGGGCGATGCCCAGTTGATGCCAGGTGCTGAGCG
CCTTCTACGACATTTGCACGCGAACAAAGTGCCTTTTGCCCTGGCCACCAGCTCCGGCG
CCGAGATGGTCGAGCTCAAGAGCACGCATCATCGTGAGCTCTTCAATCTGTTCCATCAT
CGCGTGTGCGGCTCCTCGGACAGCGAAGTGAAGAATGGCAAGCCGGCACCAGATATCT
TTTGGTGGCCGCCAGCCGTTTTGGCGATAAGCCCGAGCCGAAAAACTGTCTGGTGTTC
GAGGATTCGCCGAATGGCGTTGAGGCGGGCAACAGCGCCGGCATGCAGGTGGTCATGG
TGCCCGACGAACGGCTCTCGAAGGAGCGTTGCGCCCATGCCACCCAGGTGTTGCGCTCA
CTGGAGGACTTTAAGCCGGAACAGTTCGGCTTGCCGCCATTTACTAATTAA

MAQKVLRNVTHCIFDMDGLLLDTETLYTKAAQMVLDPYGKTYTFDVKQQIMGLQTRPVA
EFMIKCYDLPMTWEEYAKQQTDNARALMGDAQLMPGAERLLRHLHANKVPFALATSSGA
EMVELKSTHHRELFNLFHHRVCGSSDSEVKNGKPAPDIFLVAASRFGDKPEPKNCLVFEDSP
NGVEAGNSAGMQVVMVPDERLSKERCAHATQVLRSLEDFKPEQFGLPPFTN-

>Exon 1/3/4; exon 2 missing (T2)
ATGGCTCAGAAGGTATTGCGAAATGTGACGCACTGCATCTTTGACATGGATGGCCTGCT
GCTGGACACTGAACGCATTTACGAGGAGGTCACCAGGCAAATAGCTGGCACTTTCAATC
GCCCCTACCCCGAGGTGGTGCGCTTCAGAGTGATGGGCACTACGGAACAGCGCTCCGCC
GAAATAGCCGTCAATGAATGCCAATTGCCCATAACCGTCGGTGATTTTCTGCAGCGCTA
TCACAAAATGTGCTGCGAACGGCTGCACAATGTGCCGCTGCTGAAAGGTGCTGAGCGCC
TTCTACGACATTTGCACGCGAACAAAGTGCCTTTTGCCCTGGCCACCAGCTCCGGCGCC
GAGATGGTCGAGCTCAAGAGCACGCATCATCGTGAGCTCTTCAATCTGTTCCATCATCG
CGTGTGCGGCTCCTCGGACAGCGAAGTGAAGAATGGCAAGCCGGCACCAGATATCTTTT
TGGTGGCCGCCAGCCGTTTTGGCGATAAGCCCGAGCCGAAAAACTGTCTGGTGTTCGAG
GATTCGCCGAATGGCGTTGAGGCGGGCAACAGCGCCGGCATGCAGGTGGTCATGGTGC
CCGACGAACGGCTCTCGAAGGAGCGTTGCGCCCATGCCACCCAGGTGTTGCGCTCACTG
GAGGACTTTAAGCCGGAACAGTTCGGCTTGCCGCCATTTACTAATTAA

MAQKVLRNVTHCIFDMDGLLLDTERIYEEVTRQIAGTFNRPYPEVVRFRVMGTTEQRSAEIA
VNECQLPITVGDFLQRYHKMCCERLHNVPLLKGAERLLRHLHANKVPFALATSSGAEMVEL
KSTHHRELFNLFHHRVCGSSDSEVKNGKPAPDIFLVAASRFGDKPEPKNCLVFEDSPNGVEA
GNSAGMQVVMVPDERLSKERCAHATQVLRSLEDFKPEQFGLPPFTN-

>Exon 1-4, all exons (T3)
ATGGCTCAGAAGGTATTGCGAAATGTGACGCACTGCATCTTTGACATGGATGGCCTGCT
GCTGGACACTGAGACCCTCTACACGAAGGCCGCCCAAATGGTGCTCGATCCGTATGGCA
AGACGTACACGTTCGATGTGAAGCAGCAGATAATGGGTCTGCAGACGCGTCCGGTGGC
CGAGTTCATGATTAAATGCTACGACCTACCGATGACTTGGGAAGAGTACGCCAAACAAC
AGACGGACAATGCACGCGCCCTCATGGGCGATGCCCAGTTGATGCCAGACACTGAACG
CATTTACGAGGAGGTCACCAGGCAAATAGCTGGCACTTTCAATCGCCCCTACCCCGAGG
TGGTGCGCTTCAGAGTGATGGGCACTACGGAACAGCGCTCCGCCGAAATAGCCGTCAAT
GAATGCCAATTGCCCATAACCGTCGGTGATTTTCTGCAGCGCTATCACAAAATGTGCTG
CGAACGGCTGCACAATGTGCCGCTGCTGAAAGGTGCTGAGCGCCTTCTACGACATTTGC

ACGCGAACAAAGTGCCTTTTGCCCTGGCCACCAGCTCCGGCGCCGAGATGGTCGAGCTC
AAGAGCACGCATCATCGTGAGCTCTTCAATCTGTTCCATCATCGCGTGTGCGGCTCCTCG
GACAGCGAAGTGAAGAATGGCAAGCCGGCACCAGATATCTTTTTGGTGGCCGCCAGCC
GTTTTGGCGATAAGCCCGAGCCGAAAAACTGTCTGGTGTTCGAGGATTCGCCGAATGGC
GTTGAGGCGGGCAACAGCGCCGGCATGCAGGTGGTCATGGTGCCCGACGAACGGCTCT
CGAAGGAGCGTTGCGCCCATGCCACCCAGGTGTTGCGCTCACTGGAGGACTTTAAGCCG
GAACAGTTCGGCTTGCCGCCATTTACTAATTAA

MAQKVLRNVTHCIFDMDGLLLDTETLYTKAAQMVLDPYGKTYTFDVKQQIMGLQTRPVA
EFMIKCYDLPMTWEEYAKQQTDNARALMGDAQLMPDTERIYEEVTRQIAGTFNRPYPEVV
RFRVMGTTEQRSAEIAVNECQLPITVGDFLQRYHKMCCERLHNVPLLKGAERLLRHLHANK
VPFALATSSGAEMVELKSTHHRELFNLFHHRVCGSSDSEVKNGKPAPDIFLVAASRFGDKPE
PKNCLVFEDSPNGVEAGNSAGMQVVMVPDERLSKERCAHATQVLRSLEDFKPEQFGLPPFT
N-

>exon 1-4 + I; extra intron 2 (T1)
ATGGCTCAGAAGGTATTGCGAAATGTGACGCACTGCATCTTTGACATGGATGGCCTGCT
GCTGGACACTGAGACCCTCTACACGAAGGCCGCCCAAATGGTGCTCGATCCGTATGGCA
AGACGTACACGTTCGATGTGAAGCAGCAGATAATGGGTCTGCAGACGCGTCCGGTGGC
CGAGTTCATGATTAAATGCTACGACCTACCGATGACTTGGGAAGAGTACGCCAAACAAC
AGACGGACAATGCACGCGCCCTCATGGGCGATGCCCAGTTGATGCCAGGTACTAATTTT
TGATTCCGTATTCGCACTTTCCTTTCAGACACTGAACGCATTTACGAGGAGGTCACCAGG
CAAATAGCTGGCACTTTCAATCGCCCCTACCCCGAGGTGGTGCGCTTCAGAGTGATGGG
CACTACGGAACAGCGCTCCGCCGAAATAGCCGTCAATGAATGCCAATTGCCCATAACCG
TCGGTGATTTTCTGCAGCGCTATCACAAAATGTGCTGCGAACGGCTGCACAATGTGCCG
CTGCTGAAAGGTGCTGAGCGCCTTCTACGACATTTGCACGCGAACAAAGTGCCTTTTGC
CCTGGCCACCAGCTCCGGCGCCGAGATGGTCGAGCTCAAGAGCACGCATCATCGTGAGC
TCTTCAATCTGTTCCATCATCGCGTGTGCGGCTCCTCGGACAGCGAAGTGAAGAATGGC
AAGCCGGCACCAGATATCTTTTTGGTGGCCGCCAGCCGTTTTGGCGATAAGCCCGAGCC
GAAAAACTGTCTGGTGTTCGAGGATTCGCCGAATGGCGTTGAGGCGGGCAACAGCGCC
GGCATGCAGGTGGTCATGGTGCCCGACGAACGGCTCTCGAAGGAGCGTTGCGCCCATGC
CACCCAGGTGTTGCGCTCACTGGAGGACTTTAAGCCGGAACAGTTCGGCTTGCCGCCAT
TTACTAATTAA

MAQKVLRNVTHCIFDMDGLLLDTETLYTKAAQMVLDPYGKTYTFDVKQQIMGLQTRPVA
EFMIKCYDLPMTWEEYAKQQTDNARALMGDAQLMPGTNF-
FRIRTFLSDTERIYEEVTRQIAGTFNRPYPEVVRFRVMGTTEQRSAEIAVNECQLPITVGDFLQ
RYHKMCCERLHNVPLLKGAERLLRHLHANKVPFALATSSGAEMVELKSTHHRELFNLFHH
RVCGSSDSEVKNGKPAPDIFLVAASRFGDKPEPKNCLVFEDSPNGVEAGNSAGMQVVMVP
DERLSKERCAHATQVLRSLEDFKPEQFGLPPFTN-

Figure S2. Nucleotide and amino acid sequences of different *Gs1l* splice variants from Fairbanks flies. Variant numbers corresponding to those from the Korpilahti transcriptome data (see Figure S3) are given in parentheses. Intron 2 area included in variant T1 is marked with grey shading.

Fig. S3

Exons 1/3/4	MAQKVLRNVTHCIFDMDGLLL---------------------------------------
Exons 1/2/4	**MAQKVLRNVTHCIFDMDGLLL**DTETLYTKAAQMVLDPYGKTYTFDVKQQIMGLQTRPVAE
Exons 1/2/3/4 + I	**MAQKVLRNVTHCIFDMDGLLL**DTETLYTKAAQMVLDPYGKTYTFDVKQQIMGLQTRPVAE
Exons 1/2/3/4	**MAQKVLRNVTHCIFDMDGLLL**DTETLYTKAAQMVLDPYGKTYTFDVKQQIMGLQTRPVAE
Exons 1/3/4	---DTERIYEEVTRQI
Exons 1/2/4	FMIKCYDLPMTWEEYAKQQTDNARA**LMGDAQLMP**--------------------------
Exons 1/2/3/4 + I	FMIKCYDLPMTWEEYAKQQTDNARA**LMGDAQLMP**GTNF-FRIRTFLS**DTERIYEEVTRQI**
Exons 1/2/3/4	FMIKCYDLPMTWEEYAKQQTDNARA**LMGDAQLMP**-------------**DTERIYEEVTRQI**
Exons 1/3/4	AGTFNRPYPEVVRFRVMGTTEQRSAEIAVNECQLPITVGDFLQRYHKMCCERLHNVPLLK
Exons 1/2/4	--
Exons 1/2/3/4 + I	**AGTFNRPYPEVVRFRVMGTTEQRSAEIAVNECQLPITVGDFLQRYHKMCCERLHNVPLLK**
Exons 1/2/3/4	**AGTFNRPYPEVVRFRVMGTTEQRSAEIAVNECQLPITVGDFLQRYHKMCCERLHNVPLLK**
Exons 1/3/4	GAERLLRHLHANKVPFALATSSGAEMVELKSTHHRELFNLFHHRVCGSSDSEVKNGKPAP
Exons 1/2/4	**GAERLLRHLHANKVPFALATSSGAEMVELKSTHHRELFNLFHHRVCGSSDSEVKNGKPAP**
Exons 1/2/3/4 + I	**GAERLLRHLHANKVPFALATSSGAEMVELKSTHHRELFNLFHHRVCGSSDSEVKNGKPAP**
Exons 1/2/3/4	**GAERLLRHLHANKVPFALATSSGAEMVELKSTHHRELFNLFHHRVCGSSDSEVKNGKPAP**
Exons 1/3/4	DIFLVAASRFGDKPEPKNCLVFEDSPNGVEAGNSAGMQVVMVPDERLSKERCAHATQVLR
Exons 1/2/4	**DIFLVAASRFGDKPEPKNCLVFEDSPNGVEAGNSAGMQVVMVPDERLSKERCAHATQVLR**
Exons 1/2/3/4 + I	**DIFLVAASRFGDKPEPKNCLVFEDSPNGVEAGNSAGMQVVMVPDERLSKERCAHATQVLR**
Exons 1/2/3/4	**DIFLVAASRFGDKPEPKNCLVFEDSPNGVEAGNSAGMQVVMVPDERLSKERCAHATQVLR**
Exons 1/3/4	SLEDFKPEQFGLPPFTN-
Exons 1/2/4	**SLED**FKPEQFGLPPFTN-
Exons 1/2/3/4 + I	**SLED**FKPEQFGLPPFTN-
Exons 1/2/3/4	**SLED**FKPEQFGLPPFTN-

Figure S3. Amino acid alignment of the *GS1l* splice variants. Intron 2 area included in variant T1 is marked with grey color. Conserved amino acids in the protein domain especially at the core and metal-binding site in the variants including exon 2 are bolded (see main text for the details).

Fig. S4

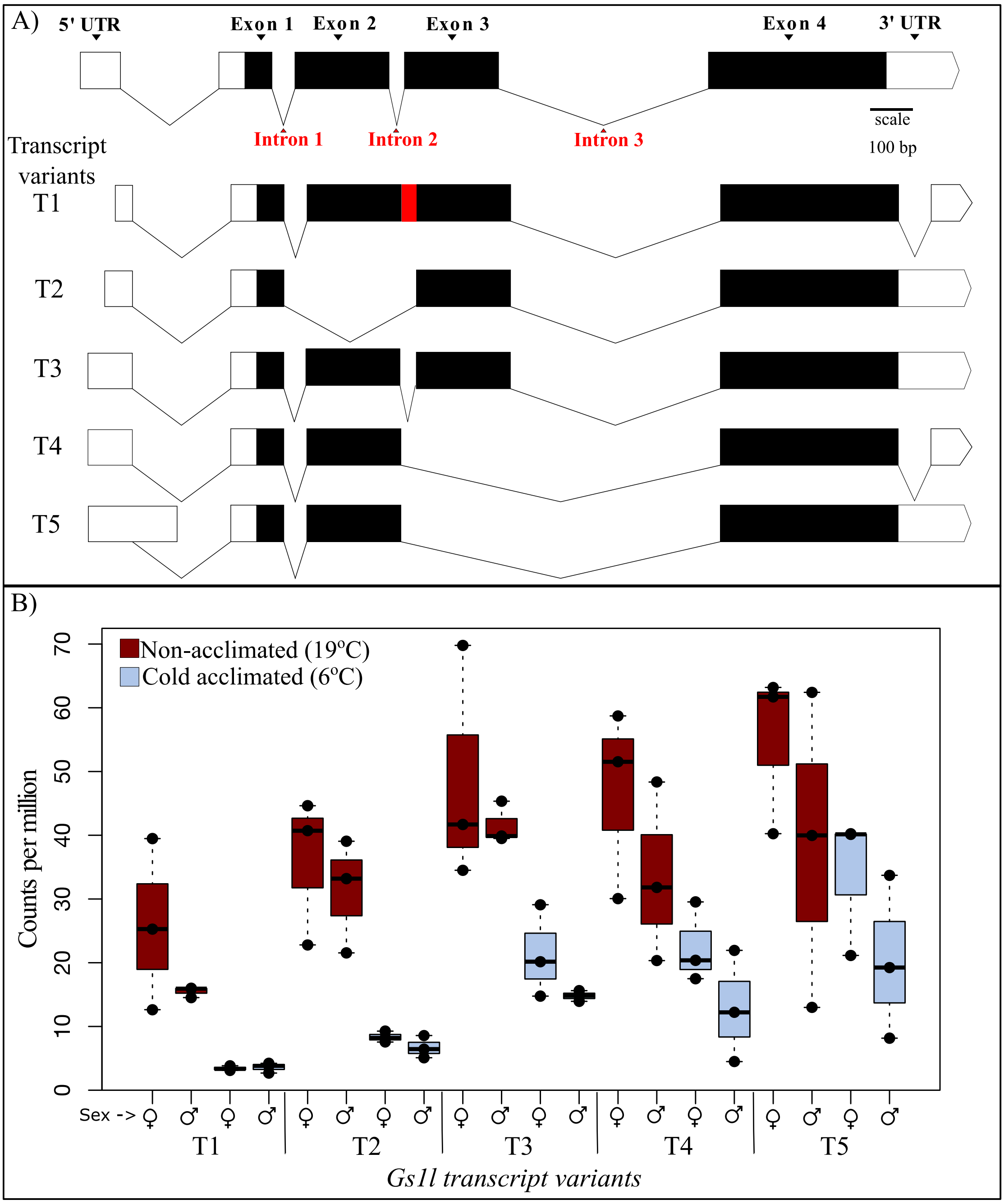

Figure S4. *Gs1l* splice variant information collected from the transcriptome data. A) Schematic presentation of the different splice variants of *Gs1l* gene. **B)** Differences in counts per million for each variant of *Gs1l* gene between non-acclimated (19˚C, red) and cold acclimated (6˚C, blue) *D. montana* female and male flies. See Supplementary Data S1 for details of the transcriptome data analyses.

Fig. S5

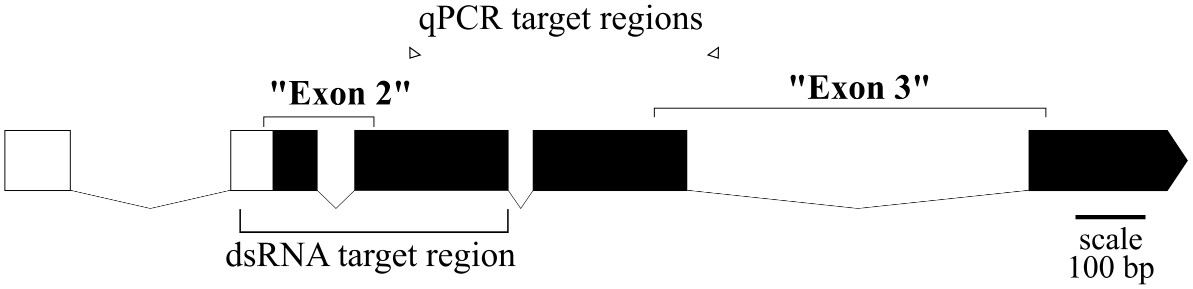

Figure S5. qPCR and dsRNA target areas in *Gs1l*. Exon 2 and exon 3 regions of the *Gs1l* gene used in the qPCR expression analysis and in the dsRNA injections (area including part of the 5’ UTR region, exon 1 and exon 2).

Fig. S6

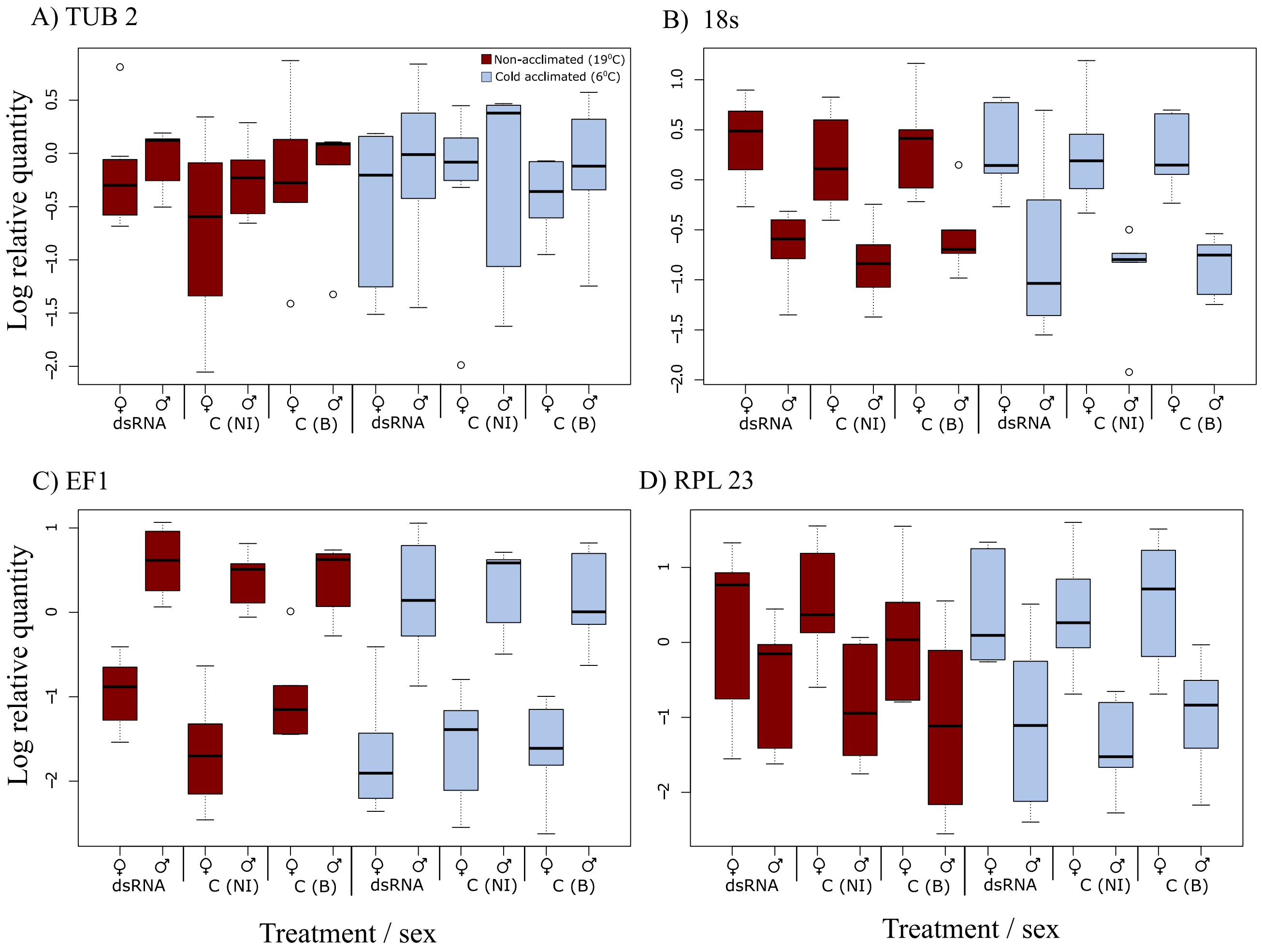

Figure S6. The relative expression values of the tested control genes in qPCR. Only *alpha-Tubulin 2* (*α-Tub 2*) had expression patterns non-biased by treatment or gender and was thus used for ΔΔ(Ct) normalization method.

Fig. S7

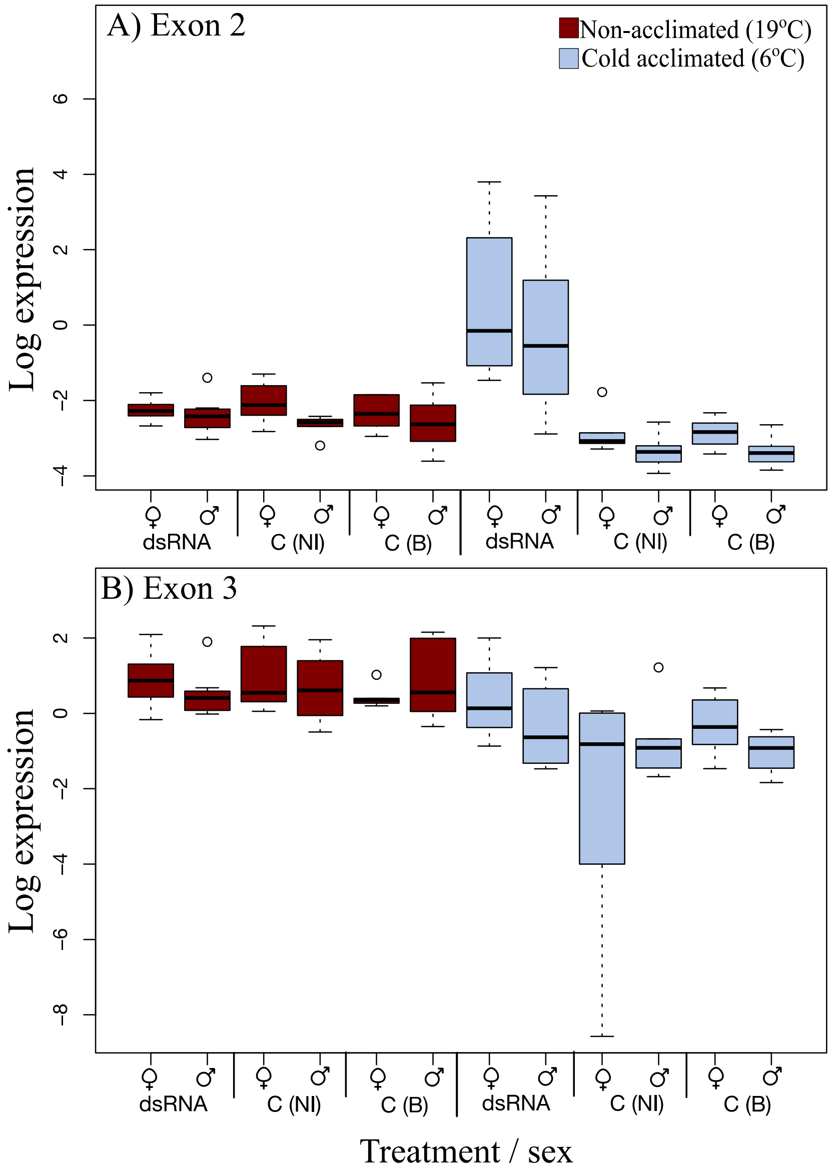

Figure S7. Normalised expression values using the ΔΔCt method and *alpha-Tubulin 2 (Tub2)* as a control gene. Log2 transformed normalized expression levels for A) exon 2 and B) exon 3 target regions of the *GS1l* gene in qPCR.

Table S1. Summary of sequence of *Gs1l* splice variant found for 12 individual flies. Splice variant were isolated using molecular cloning.

Fly sex	Treatment temperature	Injection treatment	Total number of sequences form fly	Number of short sequence with exon2	Number of short sequence with exon3	Number of long sequence with intron	Number of long sequence without intron
♀	6ºC	dsRNA	**6**	6	0	0	0
♂	6 ºC	dsRNA	**5**	5	0	0	0
♀	6 ºC	No injection	**6**	6	0	0	0
♂	6 ºC	No injection	**6**	4	0	2	0
♀	6 ºC	Buffer	**5**	3	2	0	0
♂	6 ºC	Buffer	**5**	2	2	1	0
♀	19 ºC	dsRNA	**8**	2	2	2	2
♂	19 ºC	dsRNA	**5**	4	1	0	0
♀	19 ºC	No injection	**6**	4	2	0	0
♂	19 ºC	No injection	**7**	3	2	2	0
♀	19 ºC	Buffer	**7**	2	2	2	1
♂	19 ºC	Buffer	**5**	3	2	0	0
		**Tot.**	**71**	**44**	**15**	**9**	**3**

Table S2. Sample sizes for the Critical thermal minimum (CT_{min}) analysis. Fly treatments are dsRNA injection ("dsRNA"), control with no injection ("C(NI)") and control with a Ringer buffer injection ("C(B)").

		**Cold acclimated (+6ºC)**			**Non-acclimated (+19ºC)**		
	**Sex**	**dsRNA**	**C(NI)**	**C(B)**	**dsRNA**	**C(NI)**	**C(B)**
**Ashford Population**							
Block 1	Female	15	10	14	17	9	16
	Male	18	7	15	14	9	17
Block 2	Female	21	18	17	16	18	16
	Male	21	18	17	15	18	17
**Fairbanks population**							
Block 1	Female	13	7	17	14	9	17
	Male	16	10	14	17	6	17
Block 2	Female	27	11	17	25	11	15
	Male	22	11	20	25	11	16
**Block combined**							
Ashford	Female	28	17	31	31	18	33
	Male	34	17	29	31	15	34
Fairbanks	Female	48	29	34	41	29	31
	Male	43	29	37	40	29	33

Table S3. Primers used in the study. Table gives the primer name, molecular technique it was used for and the nucleotide sequences in 5' – 3' orientation.

Primer name	Technique	Sequences 5'- 3'	
Gs1l – dsRNA target	dsRNA production	F-	ACCTGGCATCAACTGGGCATC
		R-	GTTCGCCTGTAAACTCCCCCTA
Gs1l – exon 1 to 4	molecular cloning	F-	CGAGCGAAGGAGAAGAAATG
		R-	GCGTGCAAATGTCGTAGAAG
Gs1l – exon 2	qPCR target	F-	AGAGGGTCTCAGTGTCCAGCAG
		R-	TTACATAACGAGCGAAGGAGAAG
Gs1l – exon 3	qPCR target	F-	AACCGTCGGTGATTTTCTGC
		R-	CGATGATGCGTGCTCTTGAG
18S	qPCR control	F-	AATGCACCGAGGAGGAGGTTGA
		R-	CGACCTGTAGTTTTGGTGTGCTGG
RpL32	qPCR control	F-	CATCAGCAGCACCTCCAGTTC
		R-	GATATGCCAAGCTGTCGCACAA
EF1α100E	qPCR control	F-	GCCGATTTTACTGCTCAGGTAAT
		R-	ACGCATAGAGGCTTGGATGGA
α-Tub 2	qPCR control	F-	TCATTGTCGTGTGTGAAAAA
		R-	CCGAAAGAGTGGAAGATCAG

Table S4. Sample sizes for gene expression analysis of the Fairbanks flies in qPCR. Fly treatments are dsRNA injection ("dsRNA"), control with no injection ("C(N)") and control with a Ringer buffer injection ("C(B)").

	**+6°C acclimated**			**+19°C non-acclimated**		
**sex**	**dsRNA**	**C(N)**	**C(B)**	**dsRNA**	**C(N)**	**C(B)**
Female	6	6	6	8	7	6
Male	10	5	6	7	5	6
**Both**	**16**	**11**	**12**	**15**	**12**	**12**
